# Effects of vitamin D supplementation on the functional outcome in patients with osteoporotic vertebral compression fracture and vitamin D deficiency

**DOI:** 10.1186/s13018-021-02717-7

**Published:** 2021-09-24

**Authors:** Sangbong Ko, ChungMu Jun, Junho Nam

**Affiliations:** Department of Orthopaedic Surgery, School of Medicine, Daegu Catholic University, Daegu Catholic University Hospital, 33, Duryugongwon-ro 17-gil, Nam-gu, Daegu, 42472 Korea

**Keywords:** Vitamin D, Osteoporotic fracture, Vertebra, Functional outcome

## Abstract

**Background:**

In osteoporotic vertebral compression fractures, supplementation using vitamin D preparations and maintenance of blood vitamin D level within the normal range are necessary for proper fracture union, enhancement of muscle strength, and maintenance of body balance. The purpose of this study is to investigate the effects of vitamin D supplementation on blood vitamin D level, pain relief, union time, and functional outcome in patients with osteoporotic vertebral compression fracture and vitamin D deficiency.

**Methods:**

One hundred thirty patients who were deficient in blood vitamin D level and had osteoporotic vertebral compression fracture were divided into supplementation group and non-supplementation group. Initially, 3 months, 6 months, and 12 months after the injury, radiographs were taken to assess fracture union, and questionnaires were evaluated to evaluate the functional outcome and quality of life.

**Results:**

The mean age of the 130 patients (36 males and 94 females) was 74.75 ± 7.25 years. There were no statistically significant differences in initial severity of low back pain, functional outcome, and quality of life between the insufficient group and the deficient group (all *p* values were > 0.05). There was no significant time-by-group interaction between the supplementation group and the non-supplementation group (*p* = 0.194). In terms of SF-36 physical component score, there was no significant time-by-group interaction between the supplementation group and the non-supplementation group (*p* = 0.934).

**Conclusions:**

Fracture union was achieved in all patients regardless of serum vitamin D level, and there were significant improvements in severity of low back pain, functional outcome, and quality of life over 12 months in patients with osteoporotic vertebral compression fracture. Short-term vitamin D supplementation of patients with osteoporotic vertebral compression fracture and deficiency of vitamin D did not result in significant differences in fracture union status, functional outcome, and quality of life between the supplementation groups and the non-supplementation groups of patients.

## Introduction

Osteoporotic fractures in the elderly refer to pathological fractures that occur even with minor trauma as a result of a decrease in bone mineral density (BMD) and low bone quality. Vertebral fractures are the most common osteoporotic fractures, and they have various clinical courses. For example, some vertebral fractures show no symptoms and require no treatment, some others improve with conservative treatment, while some others require surgical treatment [1]. Treatment of osteoporotic vertebral compression fracture (OVCF) is important, but prioritizing the existing osteoporosis treatment to prevent OVCF is considered the most important aspect of treatment. Osteoporosis remains an under-recognized and undertreated disease entity in orthopedic settings, accounting for significant long-term morbidity and mortality [2].

Vitamin D is known to play an important role in the regulation of blood calcium level and in the maintenance of blood phosphorus level. It is also known to play a vital role in the maintenance of healthy bones. Hence, there has been increasing interest in vitamin D. Calcium and phosphorus are involved in vitamin D metabolism and in the regulation of blood calcium level by their action on the intestine and kidney. Further, vitamin D deficiency is known to be associated with fragility fractures, osteoporosis, osteoporotic fractures, muscle weakness, and body balance. Vitamin D deficiency is also known to negatively affect fracture recovery. Moreover, vitamin D supplementation has been reported to improve bone mineralization, prevent and treat osteoporosis, prevent fractures, and prevent falls by improving muscle function in patients over the age of 65 [3, 4]. However, in some systematic reviews, it was reported that vitamin D supplementation is not an effective means of improving BMD [5], preventing falls [6–9], or preventing fractures [9–12]. Other studies report that calcium and vitamin D supplementation is important for osteoporosis treatment in patients who are deficient in calcium and vitamin D. In a previous study, it was reported that 75% of patients with incident vertebral fracture did not receive calcium and vitamin D supplementation at any time during the study [2]. As shown above, vitamin D plays different roles in the treatment of OVCF, so there is no standardized guideline on the supplementation of blood vitamin D levels.

It has been hypothesized that blood vitamin D levels fall in the early stage of fracture union after an OVCF, and this fall in vitamin D levels may have some effects on fracture union. Further, supplementation using vitamin D preparations and maintenance of blood vitamin D level within the normal range are necessary for proper fracture union, enhancement of muscle strength, and maintenance of body balance. The aim of this study is to investigate the effects of vitamin D supplementation on blood vitamin D level, pain relief, union time, and functional outcome in patients with OVCF and vitamin D deficiency.

## Materials and methods

### Patient population

After approval was granted by the Institutional Review Board (approval number, CR 20-080) and informed consent was obtained from the participants, we included 130 patients with OVCF who visited our clinic between January 2019 and January 2020 in this study based on the selection criteria (Table 1). Based on self-determination of vitamin D supplementation and a full explanation of the beneficial effects and adverse effects of vitamin D supplementation (e.g., headache, asthenia, weakness, muscle ache, anorexia, nausea, weight loss, vomiting, constipation, and so on), the 130 patients were divided into a supplementation group (*n* = 65) and a non-supplementation group (*n* = 65).

**Table 1:** Selection criteria

Inclusion criteria
1. Treatment for OVCF at our clinic during the data accumulation period
2. Follow-up period > 12 months
3. Recent OVCF at ≥ 1 level on a simple radiograph and CT or MRI
4. No other abnormal findings of the spine (e.g., infection, tumor)
5. Full understanding of the study and voluntary granting of written consent
6. Low serum vitamin D level (< 30 ng/mL)
Exclusion criteria
1. Daily intake ≥ 800 IU of vitamin D3
2. Serum calcium level > 10.5 mg/dL
3. Hypercalciuria (i.e., spot urine calcium/creatinine ratio > 0.4)
4. Malabsorption disease, lymphoma, sarcoidosis, tuberculosis, hyperparathyroidism, celiac disease
5. Kidney stone
6. Renal dysfunction (GFR < 30 mL/min/1.73 m^2^)
7. Hepatic dysfunction
8. Fasting blood sugar level > 126 mg/dL
9. Previous spine surgery
10. Secondary gain such as worker compensation and traffic insurance
11. Contraindication to vitamin D supplementation

OVCF, osteoporotic vertebral compression fracture; CT, computed tomography; MRI, magnetic resonance imaging; GFR, glomerular filtration rate

### Methods

Blood vitamin D level was determined immediately after OVCF. Demographic characteristics, basic information, medical history, comorbid diseases, and previous vitamin D supplementation were checked during the initial visit, and simple radiographs (anteroposterior and lateral radiographic views of the thoracolumbar spine in standing position) were taken to confirm the fracture level, and tenderness at the fracture level was confirmed. The initial functional outcome and quality of life (QoL) were also evaluated. About a week after the injury, simple radiographs were again taken, and patients with low blood vitamin D levels were included. Following thorough explanation of the study design and vitamin D supplementation to the patients, they were divided into supplementation group (S group) and non-supplementation group (NS group). Simple radiographs were again taken between weeks 4 and 6 after the injury to confirm further collapse and fracture union.

At 3 months, 6 months, and 12 months after the injury, simple radiographs were taken to assess fracture union using the flexion–extension dynamic view, blood was repeatedly sampled at the same time as the first blood sample, serum vitamin D (25-hydroxyvitamin D or 25(OH)D) level was measured, and functional outcome and QoL were prospectively evaluated using questionnaires. The same analgesic was administered to all patients during the initial visit, and the analgesic dose was adjusted for effective pain control. Denosumab (60 mg every 6 months) and calcium (at least 1000 mg/day) were administered to all patients, and no additional bracing was performed. The S group was administered cholecalciferol 300,000 IU or 100,000 IU SQ (Abiogen Pharma, Pisa, Italy) depending on the serum 25(OH)D level and then continued to take oral vitamin D supplementation and maintained normal serum 25(OH)D level for 12 months. However, the NS group did not supplement with vitamin D even if serum 25(OH)D levels were insufficient or deficient. Serum 25(OH)D level was measured using chemiluminescence immunoassay (LIAISON-XL, DiaSorin, Inc., Stillwater, Minnesota, USA) and blood collected between 8:00 a.m. and 8:30 a.m. on an empty stomach to reduce circadian variation. Based on the 25(OH)D level, the patients were divided into the following groups: deficient or D group [25(OH)D level < 20 ng/mL (50 nmol/L)], insufficient or I group [25(OH)D level = 21–29 ng/mL], and normal group [25(OH)D level > 30 ng/mL (75 nmol/L)] [6]. Bed rest was not recommended for all patients, and walking was recommended as soon as the acute pain subsided. The average bed rest period of all patients was less than 3 days.

### Evaluation of functional outcome and QoL

The severity of low back pain (LBP) was evaluated using a visual analog scale (VAS) with levels 0 to 10. To evaluate functional outcome, the Korean version of Oswestry Disability Index (ODI, version 2.0) and Roland Morris Disability Questionnaire (RMDQ) with levels 0 to 100 were used. Short Form 36 (SF-36) was used to evaluate QoL. This evaluation was performed by a professional clinical research coordinator (LCY) before the initial treatment and at 3, 6, and 12 months after the initial treatment.

### Statistical analysis

We used IBM SPSS Statistics version 23.0 (IBM Corp., Armonk, NY, USA) for statistical analysis. Descriptive statistics such as mean ± standard deviation, median, and interquartile range were estimated. After verification of normal distribution using Kolmogorov–Smirnov test, Student’s *t *test was performed for parametric data. For nonparametric data, Mann–Whitney *U* test and the generalized estimating equation (GEE) were used. When the GEE test for repeated measures was significant, the least significant difference test was used for post hoc pairwise multiple comparisons. *p* < 0.05 was considered statistically significant.

## Results

### Epidemiological results

The mean age of the 130 patients (36 males and 94 females) was 74.75 ± 7.25 years. The mean age of the I group (7 males and 40 females) was 76.09 ± 7.66 years, and the mean age of the D group (29 males and 54 females) was 74.05 ± 7.12 years. There was no statistically significant difference in age between the two groups (*p* = 0.458). The initial severity of LBP in the I group and the D group was 8.27 ± 1.35 and 7.38 ± 1.32, respectively. There was no statistically significant difference in severity of LBP between the two groups (*p* = 0.08). Forty-one out of 83 patients in the D group and 24 out of 47 patients in the I group were in the NS group that did not receive vitamin D supplementation, and the rest were in the S group that received vitamin D supplementation. There were 10 vertebral fractures at T11, 43 vertebral fractures at T12, 31 vertebral fractures at L1, 18 vertebral fractures at L2, 10 vertebral fractures at L3, 10 vertebral fractures at L4, and 3 vertebral fractures at L5 (Table 2).

**Table 2:** Epidemiological results

	Total patients	I group	D group	*P* value
Age (years)	74.75 ± 7.25	76.09 ± 7.66	74.05 ± 7.12	0.458
Sex (male/female)	36/94	7/40	29/54	
25(OH)D level (ng/mL)	15.37 ± 8.64	25.93 ± 3.12	9.84 ± 8.64	
Initial severity of LBP (using VAS)	7.69 ± 1.38	8.27 ± 1.35	7.38 ± 1.32	0.08
Initial ODI	25.13 ± 10.31	28.73 ± 8.40	23.24 ± 10.89	0.16
Initial RMDQ	14.28 ± 5.38	15.73 ± 4.84	13.52 ± 5.60	0.28
Initial SF-36 PCS	18.84 ± 17.54	19.26 ± 14.03	18.62 ± 19.45	0.92
Initial SF-36 MCS	26.77 ± 21.73	24.83 ± 22.23	27.28 ± 21.94	0.72
Fracture level	T11	4	6	
	T12	17	26	
	L1	8	28	
	L2	6	12	
	L3	6	4	
	L4	3	7	
	L5	3	0	

I group, insufficient group; D group, deficient group; 25(OH)D, 25-hydroxyvitamin D; LBP, low back pain; VAS, visual analog scale; ODI, Oswestry Disability Index; RMDQ, Roland Morris Disability Questionnaire; SF, short form; PCS, physical component score; MCS, mental component score

### Results of initial evaluation of functional outcome and QoL

Following evaluation of functional outcome, we found the initial ODI to be 25.13 ± 10.31 (28.73 ± 8.40 in the I group and 23.24 ± 10.89 in the D group) and the initial RMDQ level to be 14.28 ± 5.38 (15.73 ± 4.84 in the I group and 13.52 ± 5.60 in the D group). With regard to the QoL evaluation, we found that the initial SF-36 physical component score (PCS) was 18.84 ± 17.54 (19.26 ± 14.03 in the I group and 18.62 ± 19.45 in the D group) and the initial SF-36 mental component score (MCS) was 26.77 ± 21.73 (24.83 ± 22.23 in the I group and 27.28 ± 21.94 in the D group). There were no statistically significant differences in initial severity of LBP, functional outcome, and QoL between the I group and the D group (i.e., all *p* values were > 0.05) (Table 2).

### Laboratory and clinical results

In 4 patients in the D group (with initial 25(OH)D levels of 7 ng/mL, 7.3 ng/mL, 9.9 ng/mL, and 13.9 ng/mL), the serum 25(OH)D level did not return to normal even after 3 months of vitamin D supplementation (25(OH)D levels at 3 months being 16.9 ng/mL, 18.2 ng/mL, 18.4 ng/mL, and 19.4 ng/mL, respectively). However, the serum 25(OH)D levels of all 4 patients returned to normal after 6 months of vitamin D supplementation. In 1 patient in the I group, despite vitamin D supplementation, serum 25(OH)D level decreased from an initial 24.5 ng/mL to 18.4 ng/mL after 3 months but returned to normal after 6 months. The T score of the bone mineral density of all patients was -2.5 or less. Fracture union was observed in all 65 patients after about 3 months of vitamin D supplementation regardless of initial serum 25(OH)D level. There was no statistically significant relationship between serum 25(OH)D level and severity of LBP at 3 months (*p* = 0.667).

### Correlations of functional outcome and QoL in the S group and NS group

Statistically significant improvement in ODI was observed at all measurement periods in the S group, and significant improvement was observed in the NS group in all measurement periods except at 6 months. There was no statistically significant difference in the time-by-group interaction between the groups (*p* = 0.144). Statistically significant improvement in RMDQ level was observed in the S group at all measurement periods. In the NS group, no improvement in RMDQ level was observed until the initial 3 months, but statistically significant improvement was observed until the next 12 months. There was no significant time-by-group interaction between the groups (*p* = 0.194) (Table 3).

**Table 3:** Correlation between S group and NS group in ODI and RMDQ

Period	Functional outcome	Group		*P* for group differences
		S group (*n* = 65)	NS group (*n* = 65)	
Initial^a^	ODI	24.88 ± 10.46	26.40 ± 10.50	0.745
	RMDQ	14.40 ± 5.38	13.60 ± 5.89	0.753
3 mon^b^	ODI	21.07 ± 7.28	21.00 ± 13.20	0.989
	RMDQ	11.96 ± 4.22	14.20 ± 4.08	0.219
6 mon^c^	ODI	16.92 ± 7.95	19.80 ± 12.87	0.592
	RMDQ	9.11 ± 4.58	9.40 ± 5.36	0.901
12 mon^d^	ODI	14.66 ± 10.23	12.20 ± 8.55	0.530
	RMDQ	7.07 ± 5.14	6.2 ± 4.20	0.653
P for time differences	ODI	*a* > *b* > *c* > *d* (< 0.05)*	*b* > *c* (0.233) *a* > *b* > *d* (< 0.05)* *a* > *c* > *d* (< 0.05)*	Time × group *p* = 0.144
	RMDQ	*a* > *b* > *c* > *d* (< 0.05)*	*b* > *a* (0.811) *a* > *c* > *d* (< 0.05)* b > c > d (< 0.05)*	Time × group *p* = 0.194

Data were expressed as mean ± standard deviation

S group, supplementation group; NS group, non-supplementation group; ODI, Oswestry Disability Index; RMDQ, Roland Morris Disability Questionnaire

^a^Initial

^b^3 Months

^c^6 Months

^d^12 Months

^*^Statistically significant with *p* < 0.05

Statistically significant improvement in SF-36 PCS was observed at all measurement periods in the S group and the NS group. Further, in terms of SF-36 PCS, there was no significant time-by-group interaction between the groups (*p* = 0.934). Statistically significant improvement in SF-36 MCS was observed at all measurement periods in the S group, and in the NS group, significant improvement in SF-36 MCS was observed at all measurement periods except at 12 months (*p* = 0.093) (Table 4).

**Table 4:** Correlation between S group and NS group in SF-36 PCS and SF-36 MCS

Period	QoL	S group (*n* = 65)	NS group (*n* = 65)	*P* for group differences
Initial^a^	SF-36 PCS	18.69 ± 18.18	19.63 ± 15.30	0.894
	SF-36 MCS	27.59 ± 22.19	22.34 ± 20.66	0.571
6 mon^b^	SF-36 PCS	33.16 ± 19.17	32.62 ± 25.20	0.960
	SF-36 MCS	46.00 ± 21.75	52.64 ± 25.47	0.546
12 mon^c^	SF-36 PCS	50.87 ± 24.06	49.52 ± 26.75	0.908
	SF-36 MCS	59.44 ± 19.40	54.66 ± 18.76	0.567
P for time differences	SF-36 PCS	*a* < *b* < *c* (< 0.05)*	*a* < *b* < *c* (< 0.05)*	
	SF-36 MCS	*a* < *b* < *c* (< 0.05)*	*a* < *b* (< 0.001)* *a* < *c* (< 0.001)* *b* < *c* (0.662)	

Source		Group	Time	Time × group
*P* value	SF-36 PCS	0.973	< 0.001	0.934
	SF-36 MCS	0.901	< 0.001	0.093

Data were expressed as mean ± standard deviation

S group, supplementation group; NS group, non-supplementation group; QoL, quality of life; SF-36, short form 36; PCS, physical component score; MCS, mental component score

^a^Initial

^b^6 Months

^c^12 Months

^*^Statistically significant with *p* < 0.05

## Discussion

Vitamin D deficiency can lead to low BMD and an increased risk of falls and OVCF [13, 14]. Although the effect of vitamin D supplementation on the rate of OVCF has been investigated in studies [15], no study has investigated the effects of vitamin D supplementation after the onset of OVCF. A benign natural history has long been assumed for OVCFs, but up to 30% of symptomatic patients who seek treatment do not respond well to nonsurgical treatment [16, 17]. Cooper et al. reported that patient population studies suggest a positive correlation between mortality rate and number of involved vertebrae in patients with OVCF [18]. Once OVCF is diagnosed, nonsurgical management with activity modification and symptomatic medication, with or without bracing, is adequate for most patients [19], but the primary goal of OVCF management is early pain control and improvement in functional outcome.

According to a study by Barton et al., only 14% of patients who visited the emergency room for OVCF claimed to take calcium and vitamin D supplementation before their incident OVCF [2]. It was also reported that 9% of patients without prior supplementation had received vitamin D supplementation 1 year after OVCF. The study also reported that 75% of patients with incident vertebral fracture did not receive calcium and vitamin D supplementation at any time during the study [2].

Calcium and vitamin D supplementation remains a mainstay in the treatment and prevention of osteoporosis [19]. It is important in the treatment of patients with osteoporosis and deficiency of calcium and vitamin D. Vitamin D is known to play an important role in the immune system and bone health of patients with OVCF [20]. According to Kroner et al., immune cells are regulated by 1,25-hydroxyvitamin D (1,25(OH)D), and immune cells metabolically participate in the production of 1,25(OH)D from serum 25(OH)D [21]. This highlights the importance of vitamin D in shaping immune response. Vitamin D is even considered a hormone rather than a vitamin because vitamin D and its receptor are found on the surface of many other cells [22].

In many clinical trials of anti-osteoporosis drugs, vitamin D and/or calcium supplementation is administered to study participants to improve the efficacy of the anti-osteoporosis drugs [23–27]. Particularly, bisphosphonates are one of the anti-resorptive agents to treat osteoporosis. Vitamin D repletion was necessary, and vitamin D supplementations were given as an adjuvant treatment. In addition, it has been confirmed that to maximize the efficacy of anti-resorptive osteoporotic treatment and anti-fracture effect, supplementation of vitamin D for vitamin D-deficient patients is mandatory [28]. However, there is no consensus on the appropriate dose of vitamin D and calcium, but it varies from 0 to 1200 IU/day for vitamin D and from 200 to 1500 mg/day for calcium [23–27]. This dose range is appropriate for fracture prevention, but the appropriate dose after OVCF and the underlying mechanism are unknown. In this study, we aimed to determine the effect of vitamin D after OVCF. In the patient group that received supplementation of vitamin D (at least 400 IU/day) and calcium (at least 600 mg/day), the baseline sufficiency status of 25(OH)D in the blood was associated with the effect of Denosumab on BMD and fracture prevention. The study by Sugimoto et al. showed that Denosumab, which was used as a prophylactic drug for osteoporosis, did not affect the baseline vitamin D status [29]. Esposti et al. reported that the group of patients receiving calcium/vitamin D supply in addition to osteoporosis drugs had a lower risk of both subsequent fracture and all-cause mortality during the 3-year follow-up in cohort of osteoporosis patients with a recent fracture [30].

There are no other studies that have investigated the effects of vitamin D supplementation after the onset of OVF on fracture union, functional outcome, incidence of nonunion, and so on. Brinker et al. said vitamin D deficiency can adversely affect fracture healing and contribute to the development of nonunions [31]. However, a high-dose vitamin D bolus during the acute convalescence did not affect the union rate in vitamin D-deficient patients with long-bone fracture [32]. Chevalley et al. recently reported that vitamin D may have a positive influence on fracture healing and adequate vitamin D status plays an important role in the functional recovery after fracture, but the mechanism and the magnitude of the effect remain to be determined [33]. Anderson said vitamin D promotes mineralization and bone repair process [34]. There is still no consensus on what is the best supplementation strategy, in terms of dosage, frequency of treatment, and duration and even in terms of fracture union rate, mineralization process, union time, and complication rate in OVCF. A large randomized controlled study is needed to confirm the effects of vitamin D supplementation after the onset of OVCF.

The limitations of this study are presented below. First, the biggest drawback is that the sample size is little statistical significant. Although there is a group of 65 patients each, the statistical evidence for 65 patients is insufficient; it is believed that better results can be obtained if additional large-sample studies are conducted. Second, in this study, we planned vitamin D supplementation based on random extraction from the initial vitamin D deficiency group; however, we did not find relevant previous studies. Therefore, we decided to conduct a pilot study. If a randomized double-blind study based on this study is conducted in the future, more accurate results can be expected. Third, the follow-up period of 12 months is short, and there was limited information on long-term results when the baseline sufficiency status of vitamin D was maintained. Fourth, we did not consider analysis of patients’ individual diets that may result in statistical deviations. In conclusion, fracture union was achieved in all patients regardless of 25(OH)D level, and there were significant improvements in severity of LBP, functional outcome, and QoL over 12 months in patients with OVCF. Short-term vitamin D supplementation of patients with OVCF and deficiency of 25(OH)D did not result in significant differences in fracture union status, functional outcome, and QoL between the S groups and NS groups of patients. Based on the results of this study, the effects of vitamin D supplementation in patients with OVCF will be more clearly observed in a study with a larger sample size and a longer follow-up period.

## Data Availability

Available.

## Abbreviations

BMD: Bone mineral density
OVCF: Osteoporotic vertebral compression fracture
QoL: Quality of life
S group: Supplementation group
NS group: Non-supplementation group
25(OH)D: 25-Hydroxyvitamin D
D group: Deficient group
I group: Insufficient group
LBP: Low back pain
VAS: Visual analog scale
ODI: Oswestry Disability Index
RMDQ: Roland Morris Disability Questionnaire
SF-36: Short Form 36
GEE: Generalized estimating equation
PCS: Physical component scale
MCS: Mental component scale
1,25(OH)D: 1,25-Hydroxyvitamin D

## References

[1] Cho MJ, Moon SH, Lee JH (2021). Association between osteoporotic vertebral compression fractures and age, BMD, and EQ-5D in postmenopausal women in Korean orthopaedic outpatient clinics: a nationwide, observational, and cross-sectional study. Clin Orthop Surg.

[2] Barton DW, Behrend CJ, Carmouche JJ (2019). Rates of osteoporosis screening and treatment following vertebral fracture. Spine.

[3] No authors listed. Consensus development conference: diagnosis, prophylaxis, and treatment of osteoporosis. Am J Med. 1993; 94:646–50. 10.1016/0002-9343(93)90218-e 8506892

[4] Boonen S, Bischoff-Ferrari HA, Cooper C (2006). Addressing the musculoskeletal components of fracture risk with calcium and vitamin D: a review of the evidence. Calcif Tissue Int.

[5] Reid IR, Bolland MJ, Grey A (2014). Effects of vitamin D supplements on bone mineral density: a systematic review and meta-analysis. Lancet.

[6] Cameron ID, Gillespie LD, Robertson MC (2012). Interventions for preventing falls in older people in care facilities and hospitals. Cochrane Database Syst Rev.

[7] Gillespie LD, Robertson MC, Gillespie WJ (2012). Interventions for preventing falls in older people living in the community. Cochrane Database Syst Rev.

[8] Bolland MJ, Grey A, Gamble GD (2014). Vitamin D supplementation and falls: a trial sequential meta-analysis. Lancet Diabetes Endocrinol.

[9] Theodoratou E, Tzoulaki I, Zgaga L (2014). Vitamin D and multiple health outcomes: umbrella review of systematic reviews and meta-analyses of observational studies and randomised trials. BMJ.

[10] Avenell A, Mak JCS, O’Connell D (2014). Vitamin D and vitamin D analogues for preventing fractures in post-menopausal women and older men. Cochrane Database Syst Rev.

[11] Bolland MJ, Grey A, Gamble GD (2014). The effect of vitamin D supplementation on skeletal, vascular, or cancer outcomes: a trial sequential meta-analysis. Lancet Diabetes Endocrinol.

[12] Zhao JG, Zeng XT, Wang J (2017). Association between calcium or vitamin D supplementation and fracture incidence in community-dwelling older adults: a systematic review and meta-analysis. JAMA.

[13] Lips P, Bouillon R, van Schoor NM (2010). Reducing fracture risk with calcium and vitamin D. Clin Endocrinol (Oxf).

[14] Lee JH, Kim JY, Kim JY (2021). Prevalence of and risk factors for hypovitaminosis D in patients with rotator cuff tears. Clin Orthop Surg.

[15] Dawson-Hughes B, Heaney RP, Holick MF (2005). Estimates of optimal vitamin D status. Osteoporos Int.

[16] Wasnich U (1996). Vertebral fracture epidemiology. Bone.

[17] Melton LJ, Kan SH, Wahner HW (1989). Epidemiology of vertebral fractures in women. Am J Epidemiol.

[18] Cooper C, Atkinson EJ, O’Fallon WM (1992). Incidence of clinically diagnosed vertebral compression fractures: a population based study in Rochester, Minnesota, 1985–1989. J Bone Miner Res.

[19] Kim DH, Vaccaro AR (2006). Osteoporotic compression fractures of the spine; current options and considerations for treatment. Spine J.

[20] Carlberg C (2014). The physiology of vitamin D-far more than calcium and bone. Front Physiol.

[21] Kroner JC, Sommer A, Fabri M (2015). Vitamin D every day to keep the infection away?. Nutrients.

[22] Choma TJ, Rechtine GR, McGuire RA (2015). Treating the aging spine. J Am Acad Orthop Surg.

[23] Kushida K, Fukunaga M, Kishimoto H (2004). A comparison of incidences of vertebral fracture in Japanese patients with involutional osteoporosis treated with risedronate and etidronate: a randomized, double-masked trial. J Bone Miner Metab.

[24] Black DM, Delmas PD, Eastell R (2007). Once-yearly zoledronic acid for treatment of postmenopausal osteoporosis. N Engl J Med.

[25] Silverman SL, Christiansen C, Genant HK (2008). Efficacy of bazedoxifene in reducing new vertebral fracture risk in postmenopausal women with osteoporosis: results from a 3-year, randomized, placebo-, and active-controlled clinical trial. J Bone Miner Res.

[26] Cosman F, Crittenden DB, Adachi JD (2016). Romosozumab treatment in postmenopausal women with osteoporosis. N Engl J Med.

[27] Matsumoto T, Hagino H, Shiraki M (2009). Effect of daily oral minodronate on vertebral fractures in Japanese postmenopausal women with established osteoporosis: a randomized placebo-controlled double-blind study. Osteoporos Int.

[28] Catalano A, Bellone F, Santoro D (2021). Vitamin D boosts alendronate tail effect on bone mineral density in postmenopausal women with osteoporosis. Nutrients.

[29] Sugimoto T, Matsumoto T, Hosoi T (2020). Efficacy of denosumab co-administered with vitamin D and Ca by baseline vitamin D status. J Bone Miner Metab.

[30] Esposti LD, Girardi A, Saragoni S (2018). Use of antiosteoporotic drugs and calcium/vitamin D in patients with fragility fractures: impact on re-fracture and mortality risk. Endocrine.

[31] Brinker MR, O’Connor DP, Monla YT (2007). Metabolic and endocrine abnormalities in patients with nonunion. J Orthop Trauma.

[32] Haines N, Kempton LB, Seymour RB (2017). The effect of a single early high-dose vitamin D supplement on fracture union in patients with hypovitaminosis D: a prospective randomized trial. Bone Joint J.

[33] Chevalley T, Brandi ML, Cavalier E, et al. How can the orthopedic surgeon ensure optimal vitamin D status in patients operated for an osteoporotic fracture? Osteoporosis Int Epub a head. 2021. 10.1007/s00198-021-05957-9 PMC8134831 34013461

[34] Anderson PH (2017). Vitamin D activity and metabolism in bone. Curr Osteoporos Rep.

